# Assessing Self-supervised xLSTM-UNet Architectures for Head and Neck Tumor Segmentation in MR-Guided Applications

**DOI:** 10.1007/978-3-031-83274-1_12

**Published:** 2025-03-03

**Authors:** Abdul Qayyum, Moona Mazher, Steven A. Niederer

**Affiliations:** 1 National Heart and Lung Institute, Faculty of Medicine, Imperial College London, London, UK; 2 Centre for Medical Image Computing, Department of Computer Science, University College London, London, UK

**Keywords:** xLSTM-UNet architecture, Student-teacher SSL-based models, Self-supervised learning, UxLSTM, Head and neck cancer segmentation, MRI-guided radiotherapy

## Abstract

Radiation therapy (RT) plays a pivotal role in treating head and neck cancer (HNC), with MRI-guided approaches offering superior soft tissue contrast and daily adaptive capabilities that significantly enhance treatment precision while minimizing side effects. To optimize MRI-guided adaptive RT for HNC, we propose a novel two-stage model for Head and Neck Tumor Segmentation. In the first stage, we leverage a Self-Supervised 3D Student-Teacher Learning Framework, specifically utilizing the DINOv2 architecture, to learn effective representations from a limited unlabeled dataset. This approach effectively addresses the challenge posed by the scarcity of annotated data, enabling the model to generalize better in tumor identification and segmentation. In the second stage, we fine-tune an xLSTM-based UNet model that is specifically designed to capture both spatial and sequential features of tumor progression. This hybrid architecture improves segmentation accuracy by integrating temporal dependencies, making it particularly well-suited for MRI-guided adaptive RT planning in HNC. The model’s performance is rigorously evaluated on a diverse set of HNC cases, demonstrating significant improvements over state-of-the-art deep learning models in accurately segmenting tumor structures. Our proposed solution achieved an impressive mean aggregated Dice Coefficient of 0.81 for pre-RT segments and 0.65 for mid-RT segments, underscoring its effectiveness in automated segmentation tasks. This work advances the field of HNC imaging by providing a robust, generalizable solution for automated Head and Neck Tumor Segmentation, ultimately enhancing the quality of care for patients undergoing RT. Our team name is DeepLearnAI (CEMRG). The code for this work is available at https://github.com/RespectKnowledge/SSL-based-DINOv2_Vision-LSTM_Head-and-Neck-Tumor_Segmentation.

## 1 Introduction

Radiation therapy (RT) continues to play a vital role in cancer treatment, offering an effective method for managing a broad spectrum of malignancies. Head and neck cancer (HNC) is one area where RT is highly beneficial, as precise targeting is crucial to achieving effective tumor control while minimizing damage to nearby healthy tissues. Traditionally, CT-based planning has been the standard approach in RT for identifying and mapping tumors and surrounding anatomical structures. However, recent advancements have sparked growing interest in MRI-guided RT planning, which provides several notable advantages over CT. MRI provides enhanced soft tissue contrast for improved tumor visualization and enables advanced multiparametric sequences such as diffusion-weighted imaging (DWI), which adds functional imaging capabilities to the planning process. Furthermore, MRI-Linac systems support daily adaptive RT by integrating real-time imaging with treatment delivery [1]. These capabilities together enhance the precision of tumor localization and the overall efficacy of RT.

The introduction of MRI-guided adaptive RT marks a significant leap forward in cancer therapy. This approach allows for dynamic adjustments to treatment plans based on real-time MRI scans taken during RT, enabling more tailored and responsive treatments. By continuously adapting the treatment to account for changes in the tumor and surrounding tissues, MRI-guided adaptive RT aims to enhance tumor elimination while sparing healthy tissue, potentially revolutionizing treatment practices for HNC [2].

Despite its transformative potential, several challenges accompany the widespread adoption of MRI-guided RT in clinical practice. The process generates large amounts of data, especially for HNC, where accurate tumor delineation is particularly crucial. The task of manual segmentation by clinicians, which remains the clinical standard, is labor-intensive and time-consuming [3]. Additionally, the complexity of head and neck tumors makes them some of the most difficult anatomical structures to accurately segment [4]. These hurdles have prompted the exploration of automated solutions, particularly leveraging artificial intelligence (AI) and deep learning techniques, to improve the efficiency and accuracy of tumor segmentation in MRI-guided RT planning.

Incorporating AI-driven approaches into this workflow could not only reduce the time burden on clinicians but also improve the precision of tumor segmentation, facilitating the adoption of MRI-guided adaptive RT on a broader scale. The potential for real-time, adaptive treatment adjustments is a key aspect of this evolving technology, making MRI-guided RT a promising area for future innovations in cancer care.

Medical image analysis, a cornerstone of computer-aided diagnosis, often grapples with the challenge of limited labeled data, especially for intricate 3D tasks. Self-supervised learning (SSL) has emerged as a promising approach to reduce the dependence on extensive manual annotation by leveraging vast amounts of unlabeled data. However, SSL still demands large unlabeled datasets to effectively learn meaningful feature representations. This need is further compounded by the scarcity of 3D medical data, driven by the high costs of imaging and stringent privacy concerns, resulting in most datasets containing only a few cases [5–7]. Existing segmentation methods, such as U-Nets and 3D CNNs [5], often require large, annotated datasets, which are challenging to obtain in medical imaging due to the time and expertise needed for manual labeling. These methods may also struggle with capturing temporal changes in longitudinal datasets, such as MRI scans collected before and during radiation therapy. Self-supervised learning (SSL) addresses these limitations by leveraging abundant unlabeled data to learn robust spatial and structural features, reducing reliance on labeled datasets and improving generalizability. Vision-LSTM is particularly suitable for this task as it combines spatial encoding with temporal modeling, effectively capturing the progression of tumor changes over time, making it ideal for both pre-RT and mid-RT segmentation tasks.

We introduce a straightforward, yet powerful SSL approach based on the DINOv2 framework [7], aimed at minimizing the need for extensive manual annotation in 3D medical image segmentation. Our method takes advantage of available unlabeled data to create robust pre-trained models that excel in downstream segmentation tasks. In the first stage, we adapt the DINOv2 framework, originally designed for 2D tasks, to handle the complexities of 3D medical imaging using our proposed Vision-LSTM (xLSTM) [8, 9]. This adaptation involves extending the model’s architecture and training framework to process volumetric data, enabling it to learn rich, spatially aware representations from unlabeled 3D medical images. This allows the model to capture the intricate structures inherent in 3D medical datasets.

Once pre-training is complete, the learned knowledge is transferred to a specific 3D medical image segmentation task. This is achieved by extracting the encoder from the pre-trained model and fine-tuning it alongside a task-specific decoder tailored for segmentation. The pre-trained encoder serves as a strong foundation, enriched with spatial features learned during the self-supervised phase, significantly enhancing segmentation performance on the target task. To further enhance our approach given the limitations of the labeled HNC dataset, we propose a deep learning-based model for precise segmentation of HNC structures:

1. We present a modified DINOv2 framework that incorporates the 3D Vision-LSTM (xLSTM) model for self-supervised learning in the initial phase, utilizing the limited pre-RT and mid-RT MRI Tumor datasets.
2. For the downstream task, Vision-LSTM (xLSTM) serves as the backbone, with the encoder frozen and the decoder fine-tuned to improve medical image segmentation.
3. We thoroughly evaluate the proposed solution using the pre-RT and mid-RT MRI datasets, comparing its performance against state-of-the-art deep learning models to validate its effectiveness.

This approach not only overcomes the challenges of limited labeled data but also shows promise in advancing accurate 3D medical image segmentation, particularly in the context of HNC diagnosis and treatment.

## 2 Proposed Method

### 2.1 Dataset

The dataset comprises 150 T2-weighted (T2w) MRI scans of the head and neck region collected at MD Anderson Cancer Center (MDACC) [10–12]. The images include a mix of fat-suppressed and non-fat-suppressed sequences, with all patients immobilized using a thermoplastic mask. The scans were extracted from an institutional imaging repository (Evercore) and consisted of both pre-radiation therapy (pre-RT) images, taken 1–3 weeks before RT began, and mid-radiation therapy (mid-RT) images, acquired 2–4 weeks into treatment. Each patient’s image pairs are consistently either fat-suppressed or non-fat-suppressed. The dataset includes segmentations of primary gross tumor volumes (GTVp) and metastatic lymph nodes (GTVn), with GTVp present in at most one region per patient and a variable number of GTVn. Multiple physician experts (3 to 4 observers) independently annotated the GTVp and GTVn structures on both pre-RT and mid-RT scans. Based on recent research [13], at least three annotators are recommended for reliable segmentation, which were combined using the STAPLE (Simultaneous Truth and Performance Level Estimation) algorithm to create consensus segmentations. The annotators were experienced medical doctors specializing in head and neck cancer, with access to each patient’s medical history and prior imaging, such as PET/CT scans. The final segmentations were verified by radiation oncology faculty with over 10 years of experience. In cases of significant disagreement among observers, a single contour from an expert was used. A training dataset comprising 150 cases has been made available for head and neck cancer (HNC) segmentation.

We have proposed methods to address two tasks: pre-RT MRI Tumor Segmentation and mid-RT MRI Tumor Segmentation. The first task focuses on segmenting head and neck cancer (HNC) tumor volumes from MRI scans acquired before the initiation of radiation therapy. The second task involves segmenting HNC tumor volumes from MRI scans obtained during radiation therapy, utilizing a dataset that also includes images from the pre-RT segmentation task.

### 2.2 Teacher Student SSL Model for 3D Medical Imaging

**Fig. 1.**
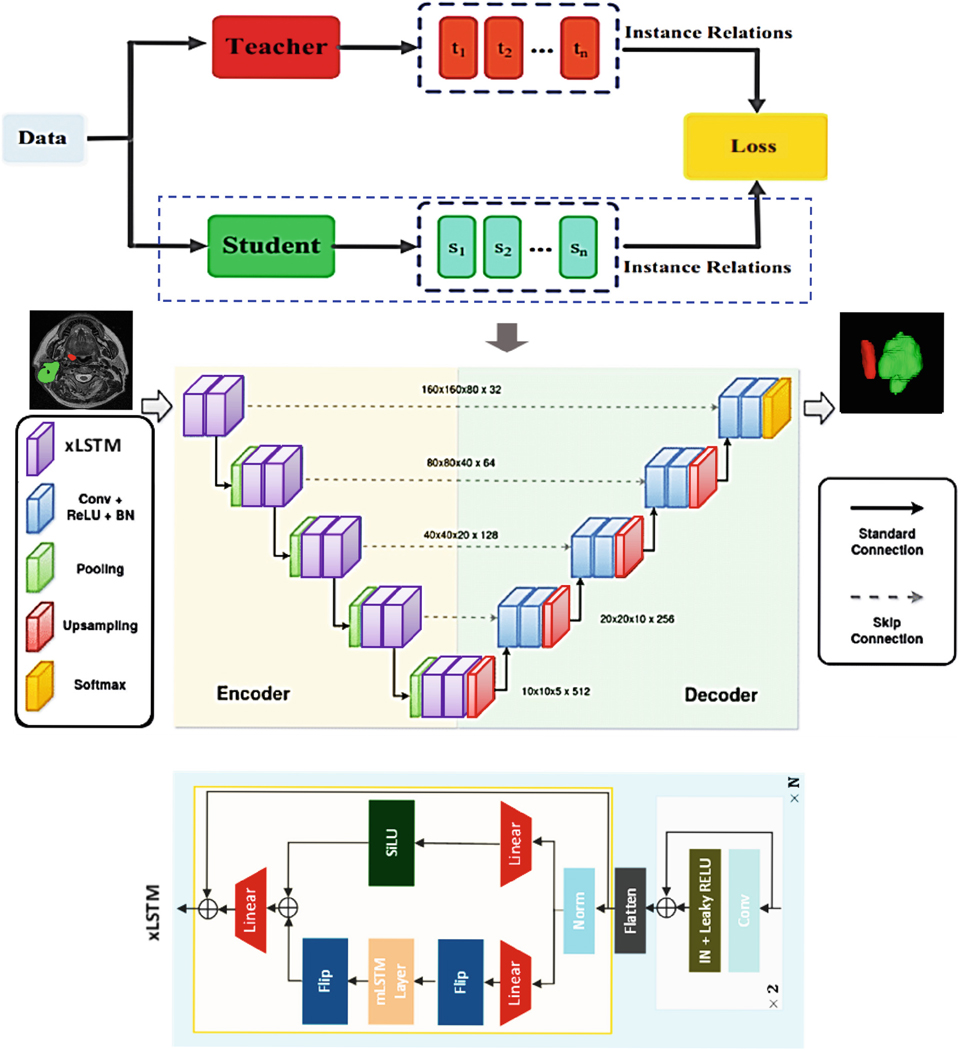
Proposed SSL model for automated Head and Neck Tumor Segmentation.

Our study is implemented using a self-supervised learning (SSL) framework that incorporates an innovative structure with a single online student encoder and a corresponding momentum-based teacher encoder. Both encoders share the same network architecture; however, the teacher encoder’s parameters are updated using momentum from the student encoder’s parameters. This setup allows the model to progressively improve its learning from unlabeled data as the teacher encoder evolves alongside the student’s performance. A key component of SSL is the use of strong data augmentations to generate diverse and informative training samples. For our study, we apply a variety of transformations to the input images, such as flipping, scaling, adding Gaussian noise and blur, and adjusting brightness and contrast. These augmentations produce two distinct views of the same input, which are processed through a Siamese network structure. By comparing these views, the model learns robust feature representations. The entire workflow is illustrated in Fig. 1.

To train the 3D Vision-LSTM (xLSTM) model, we adapted the DINOv2 approach within a self-supervised learning context. This approach enables our model to effectively leverage the unlabeled data, facilitating the learning of rich spatial representations that are essential for downstream tasks like 3D medical image segmentation. This methodology not only optimizes the use of available data but also boosts the segmentation model’s performance and resilience.

The momentum teacher encoder’s parameters $\theta_{t}$ are updated based on the student encoder’s parameters $\theta_{s}$ using a momentum-based approach:

(1) $$\theta_{t}=m\cdot \theta_{t}+(1-m)\theta_{t}$$

where $\theta_{t}$ are the parameters of the teach encoder, $\theta_{s}$ are the parameters of the student encoder, m is the momentum coefficient typically a value close to 1.

Let x be the original input image. Two different views of the input, $x_{1}$ and, $x_{2}$ are generated using strong data augmentations:

(2) $$x_{1}=Augment(x),x_{2}=Augment(x)$$

Both views are then processed through the student encoder $f_{s}$ and teacher encoder $f_{t}$ to extract feature representations:

(3) $$h_{1}=f_{s}\left(x_{1};\theta_{s}\right),h_{2}=f_{s}\left(x_{2};\theta_{s}\right)\;h_{1}^{\prime }=f_{s}\left(x_{1};\theta_{t}\right),h_{2}^{\prime }=f_{s}\left(x_{2};\theta_{t}\right)$$

where $h_{1}$ and $h_{2}$ are the feature representations from the student encoder, $h_{1}^{\prime }$ and $h_{2}^{\prime }$ are the feature representations from the teacher encoder.

The feature representations $h_{1}$, $h_{2}$, $h_{1}^{\prime }$, $h_{2}^{\prime }$ are subjected to global average pooling to reduce them into feature vectors:

(4) $$v_{1}=GAP\left(h_{1}\right),v_{2}=GAP\left(h_{2}\right)\;v_{1}^{\prime }=GAP\left(h_{1}^{\prime }\right),v_{2}^{\prime }=GAP\left(h_{2}^{\prime }\right)$$

where $v_{1}$, $v_{2}$, $v_{1}^{\prime }$ and $v_{2}^{\prime }$ are the resulting feature vectors.

(5) $$z_{1}=MLP\left(v_{1}\right),z_{2}=MLP\left(v_{2}\right)\;z_{1}^{\prime }=MLP\left(v_{1}^{\prime }\right),z_{2}^{\prime }=MLP\left(v_{2}^{\prime }\right)$$

After projection, the teacher’s output is centered, sharpened, and passed through a softmax function to produce the supervision signal:

(6) $$q_{1}^{\prime }=Softmax\left(\frac{Center\left(z_{1}^{\prime }\right)}{\tau }\right)\;q_{2}^{\prime }=Softmax\left(\frac{Center\left(z_{2}^{\prime }\right)}{\tau }\right)$$

where Center (z) subtracts the mean of the vector to have zero mean. τ is the temperature parameter controlling the sharpness of the distribution. Softmax(z) normalizes the vector into a probability distribution.

The loss function is designed to minimize the divergence between the student’s feature vectors and the teacher’s processed outputs. A common choice is the cross-entropy loss or mean squared error (MSE) between the student’s and teacher’s outputs:

(7) $$L=\frac{1}{2}\left(Loss\left(z_{1},q_{2}^{\prime }\right)+Loss\left(z_{2},q_{1}^{\prime }\right)\right)$$

where the cross-entropy loss is

(8) $$L\left(z,q^{\prime }\right)=-\sum_{k=1}^{K}q^{\prime }[k]\text{log}(Softmax(z)[k])$$

where this loss function encourages the student encoder to produce feature representations that align closely with the teacher’s outputs, thus enabling effective learning from the unlabeled data. The student model is trained to align with the sharpened output of the teacher model.

The 3D Vision-LSTM (xLSTM) model is trained within this SSL framework, where the student encoder’s parameters are updated through backpropagation to minimize the loss function L, while the teacher encoder’s parameters are adjusted using the momentum mechanism previously described. This integration of self-supervised learning, momentum-based updates for the teacher encoder, and strong data augmentation techniques forms an effective strategy for pre-training the model on unlabeled data. As a result, this approach significantly boosts the model’s performance in downstream tasks, including 3D medical image segmentation.

### 2.3 Model Architecture

The xLSTM-UNet model integrates Vision-LSTM (xLSTM), a refined version of Long Short-Term Memory (LSTM) networks. xLSTM has shown exceptional performance across various fields, such as Natural Language Processing (NLP) and image classification, surpassing models like Vision Transformers and State Space Models (SSMs) such as Mamba [8, 9]. This advanced xLSTM module is embedded within the UNet architecture, which is well-known for its ability to effectively extract local features through convolutional layers. The UNet structure is highly suited for image segmentation due to its encoder-decoder architecture, where the encoder captures hierarchical features using convolutional operations, and the decoder reconstructs and refines these features to generate segmentation maps.

### 2.4 Self-Supervised Learning (SSL) Approach

Pre-Trained Encoder: In the initial phase of our approach, we employ a self-supervised learning (SSL) technique to pre-train the encoder of the xLSTM-UNet model. This SSL framework utilizes large volumes of unlabeled data to train the encoder, focusing on the Vision-LSTM (xLSTM) model to capture rich, contextual features from the input images. This pre-training stage is essential for developing robust feature representations that generalize well across various medical imaging tasks [14].

Fine-Tuned Decoder: Following the pre-training of the encoder, we fine-tune the decoder in a supervised manner using labeled data. The decoder is designed to convert the high-level feature representations from the encoder into accurate and detailed segmentation maps. Fine-tuning the decoder’s parameters optimizes its performance for the specific segmentation task.

Enhanced Long-Range Dependency Capture: By integrating xLSTM—which excels at capturing long-range dependencies—into the UNet architecture, the model efficiently combines local feature extraction with global contextual understanding. This combination improves segmentation accuracy, particularly for complex biomedical images.

Efficient Learning with Limited Labeled Data: The SSL approach enables the effective use of unlabeled data, reducing the need for large labeled datasets. The pre-trained encoder establishes a solid foundation, while the fine-tuned decoder ensures high accuracy for targeted segmentation tasks. The xLSTM-UNet model, with its pre-trained xLSTM encoder and fine-tuned decoder, presents a powerful solution for biomedical image segmentation. This strategy not only capitalizes on advanced deep learning methods but also maximizes the utility of unlabeled data through SSL, resulting in greater accuracy and efficiency for segmentation tasks. To enhance training diversity, strong augmentations such as flipping, scaling, Gaussian noise addition and brightness adjustments are applied to create two distinct views of each input, improving the model’s robustness to data variations. In the SSL stage, the model is trained on unlabeled data for 500 epochs to learn rich feature representations, followed by fine-tuning the SSL-pretrained encoder in the segmentation stage with labeled data for 300 epochs. Input images are normalized to zero mean and unit variance, resized to a fixed resolution and processed by the 3D Vision-LSTM model. Segmentation optimization uses a combination of Dice loss and cross-entropy loss to address the class imbalance and improve pixel-wise accuracy. The Adam optimizer, with a low learning rate and scheduling adjustments, ensures stable and efficient convergence during fine-tuning. The PyTorch library is used for training, testing, and optimizing the proposed model.

## 3 Results

In this paper, we proposed a modified DINOv2 framework, incorporating the 3D Vision-LSTM (xLSTM) model for self-supervised learning, which significantly enhances medical image segmentation tasks in RT datasets. The proposed method leverages two key segmentation datasets: pre-RT segmentation (Task 1) and mid-RT segmentation (Task 2). The innovation lies in employing a self-supervised learning approach in the first stage, where the xLSTM model is utilized to process the unlabeled segmentation data, enabling the model to learn meaningful representations without the need for manual labeling. By doing this, we harness a large amount of pre- and mid-RT segmentation data to develop a robust framework for downstream tasks.

For the second stage, the xLSTM model acts as the backbone for the downstream task of medical image segmentation. Here, we freeze the encoder weights learned during the self-supervised phase and fine-tune the decoder to adapt the model to the specific segmentation tasks. This method significantly improves performance by retaining the powerful, generalized features learned during the self-supervised phase, while fine-tuning the decoder allows for the task-specific refinement required for precise segmentation of radiotherapy data.

The performance of our method was rigorously evaluated on two tasks: Task 1 (pre-RT segmentation) and Task 2 (mid-RT segmentation). The results, summarized in Table 1, demonstrate the effectiveness of our approach. For Task 1, the Dice Similarity Coefficient (DSC) achieved was 0.86045 for GTVn (Gross Tumor Volume - nodes), and 0.7690 for GTVp (Gross Tumor Volume - primary), resulting in an Aggregated DSC of 0.8147. For Task 2, which is more challenging due to the mid-RT setting, our model still performed strongly, achieving a DSC of 0.7731 for GTVn and 0.5343 for GTVp, with an Aggregated DSC of 0.6537. Compared to state-of-the-art deep learning models, our proposed framework demonstrates superior segmentation performance, particularly in Task 1, where the model showed excellent segmentation accuracy for both GTVn and GTVp. Task 2, while more challenging, also yielded competitive results, particularly in GTVn segmentation. These results highlight the robustness and generalization ability of our modified DINOv2 framework and xLSTM-based approach in handling complex medical imaging tasks, especially when processing multi-stage radiotherapy datasets. This comprehensive evaluation not only validates the efficiency of the proposed method but also underscores its potential for improving segmentation accuracy in clinical applications, particularly in radiotherapy planning, where accurate segmentation is critical for treatment success. Table 2 presents a comparison of the proposed model with state-of-the-art models, based on results from the leaderboard on two subject validation datasets. The proposed model is evaluated both with and without SSL and is also compared to the base 3D-ResUNet.

**Fig. 2.**
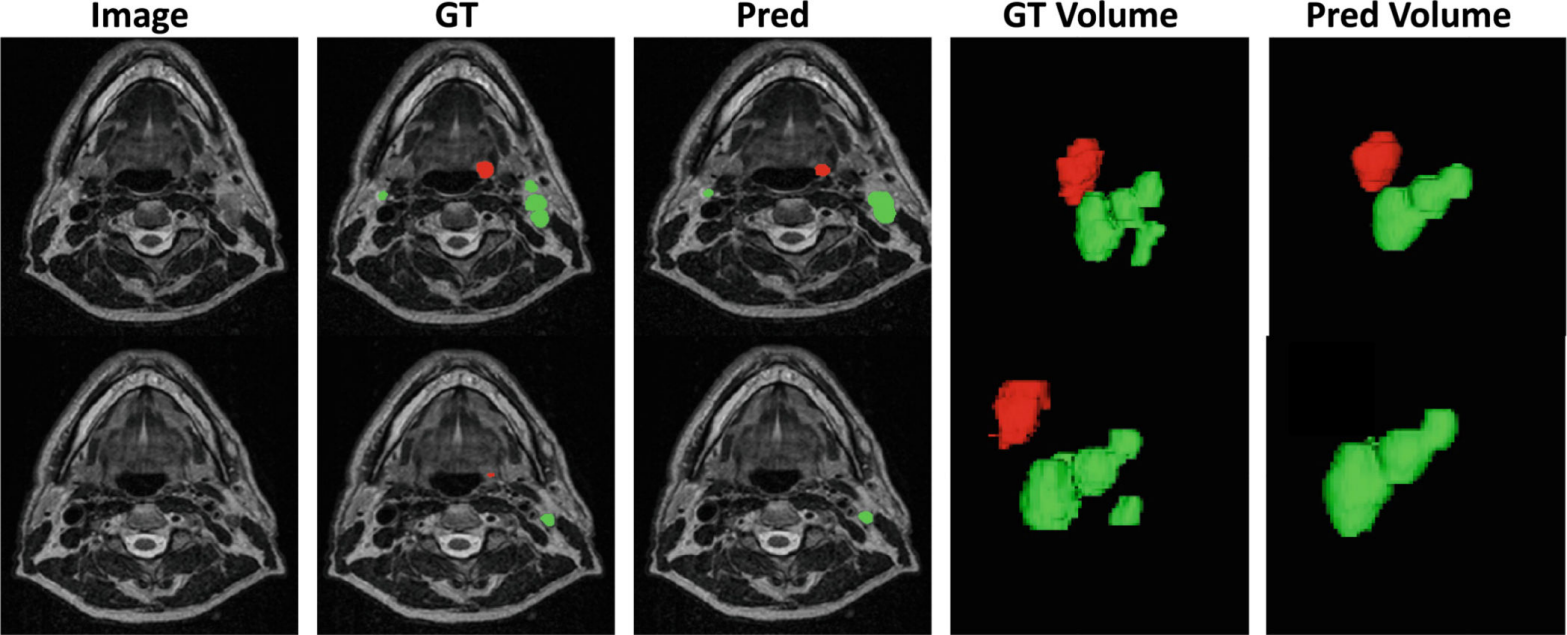
The first row displays the predictions for Task 1, while the second row presents the predictions for Task 2.

In Fig. 2, the results for two segmentation tasks are presented in two rows, each highlighting different aspects of the analysis. The first row focuses on Task 1, which involves segmenting tumor types GTVn (Gross Tumor Volume of lymph nodes) and GTVp (Gross Tumor Volume of the primary tumor). The second column shows the Ground Truth (GT) segmentation masks as a baseline for comparison, while the third column presents the segmentation results produced by the proposed model, which effectively identifies tumor regions in this task. The fourth column displays a 3D visualization of the tumor volume based on the Ground Truth (GT), providing a detailed representation of the actual tumor structure. The fifth column shows the corresponding 3D segmentation volume generated by the proposed model, which aligns well with the GT for Task 1, demonstrating the model’s capability in accurately capturing tumor features in 3D.

The second row illustrates the results for Task 2, where the performance of the proposed model is notably less accurate. As seen in the third column, the segmentation masks predicted by the model deviate significantly from the Ground Truth (GT), failing to capture the required structures effectively. Similarly, the 3D segmentation volume generated by the proposed model, shown in the fifth column, does not match the precision of the Ground Truth’s 3D representation depicted in the fourth column. These discrepancies highlight the model’s limitations in Task 2, especially in capturing complex or nuanced features, both in 2D segmentation masks and 3D volumetric reconstructions. Overall, while the proposed model performs well for Task 1, its underperformance in Task 2 underscores the need for further optimization to handle the specific challenges of this task. The relative underperformance of the proposed model in Task 2 (mid-RT segmentation) compared to Task 1 (pre-RT segmentation) can be attributed to several key factors related to the complexity of mid-RT imaging, the inherent challenges of the dataset, and the model’s adaptation capabilities.

### 1. Increased Variability in mid-RT Images

One of the primary reasons for the lower Dice Similarity Coefficient (DSC) in Task 2 is the increased variability in the imaging data during mid-radiotherapy (mid-RT). By the mid-point of radiotherapy, significant physiological changes may occur in the patient due to radiation exposure, including tumor shrinkage, changes in tissue density, and the development of inflammation or edema. These factors lead to less uniform, more complex image features that are harder for the model to segment accurately. For example, as the tumor responds to treatment, its shape, size, and boundaries may become less distinct, making it difficult for the model to differentiate between the Gross Tumor Volume of primary cancer (GTVp) and the surrounding tissue. In Task 1 (pre-RT segmentation), the tumor boundaries are typically more defined, as the imaging data is captured before any treatment effects. The model can rely on more consistent anatomical features, resulting in higher segmentation accuracy and a better DSC for both GTVn and GTVp.

### 2. Challenges in Gross Tumor Volume - Primary (GTVp) Segmentation

The model’s performance drop in Task 2 is particularly notable in the segmentation of the Gross Tumor Volume - primary (GTVp), where the DSC drops to 0.5343, compared to 0.7690 in Task 1. This indicates that GTVp segmentation is more challenging in the mid-RT phase. The mid-RT images of the primary tumor (GTVp) are affected by complex factors such as tissue deformation, changes in intensity patterns, and the presence of artifacts from ongoing radiotherapy, all of which contribute to difficulties in distinguishing the tumor from surrounding healthy or affected tissues. These variations may cause the model to misclassify or blur the boundaries between tumor and non-tumor regions, reducing its overall accuracy.

### 3 Generalization Limitations of the xLSTM Model

The xLSTM model, while effective at learning meaningful representations during the self-supervised phase in Task 1, may face challenges when generalizing to more complex scenarios in Task 2. Although the model’s encoder was frozen and only the decoder fine-tuned for the segmentation task, the features learned during the pre-RT stage might not be fully transferable to the mid-RT stage. Task 2 involves dynamic changes in the tumor and surrounding tissues that may not have been adequately represented in the pre-RT dataset, making it harder for the model to adjust effectively. The gap between the pre-RT and mid-RT domains means that features learned from the pre-RT data might not align well with the mid-RT variations, leading to reduced performance.

### 4. Impact of Limited Labelled Data

Another factor contributing to the lower performance in Task 2 is the potential limitation of labeled data available for mid-RT segmentation. Unlike pre-RT data, where the images are usually more standardized and available in larger quantities, mid-RT datasets can be scarcer and more heterogeneous, reducing the opportunity for the model to train effectively. The reduced labeled data for fine-tuning during Task 2 could limit the model’s ability to generalize well to unseen mid-RT scenarios, especially when dealing with complex tissue changes.

### 5. Balancing GTVn and GTVp Performance

In Task 2, the model performs better on GTVn segmentation (DSC: 0.7731) than GTVp (DSC: 0.5343), suggesting that the anatomical changes and treatment effects might affect the primary tumor (GTVp) more than the lymph nodes (GTVn). Lymph nodes tend to have more consistent anatomical features and may not change as drastically during treatment, allowing the model to retain higher accuracy for their segmentation. In contrast, the primary tumor, being more directly affected by radiation therapy, undergoes more pronounced changes that the model struggles to capture accurately. The lower DSC score in Task 2 compared to Task 1 reflects the greater complexity of segmenting mid-RT images, where treatment-induced anatomical and tissue changes present significant challenges for the model. These challenges, coupled with domain shift between pre- and mid-RT datasets, and potential limitations in labeled data for fine-tuning, result in a decreased ability to segment the primary tumor accurately in Task 2. However, the model still performs reasonably well on GTVn segmentation, highlighting its strengths in dealing with less variable structures like lymph nodes, while further optimization is needed to improve its robustness in handling the more complex and dynamic changes of the primary tumor during radiotherapy.

## 4 Conclusion

We have developed a two-stage approach for Pre-RT and mid-RT head and neck cancer (HNC) segmentation, incorporating advanced machine learning techniques. The method begins with a self-supervised learning phase, utilizing the DINOv2 framework and a 3D Vision-LSTM (xLSTM) model, which effectively addresses the challenges of limited data and the absence of annotated samples from the pre-RT and mid-RT HNC dataset. In the second stage, the model is fine-tuned for segmentation by freezing the encoder and optimizing the decoder, resulting in enhanced overall performance. The xLSTM model demonstrates effectiveness in accurately segmenting head and neck tumors, including GTVn (Gross Tumor Volume of lymph nodes) and GTVp (Gross Tumor Volume of the primary tumor), while also enhancing the precision of surface segmentation, as evidenced by the results. This approach offers a robust and scalable solution for automated pre-RT and mid-RT HNC segmentation, leading to more precise diagnoses and personalized treatment planning.

For future directions, we recommend expanding this approach to larger, more diverse datasets to further validate its generalizability and improve its ability to handle a wider range of cases. Integrating additional modalities, such as PET or CT scans, could enhance multimodal learning and provide more comprehensive treatment planning. Moreover, incorporating real-time feedback during treatment could further refine the accuracy of adaptive radiation therapy. By advancing this model’s application to other cancer types or anatomical regions, it could become a key tool in precision oncology, contributing to more individualized and effective cancer treatment strategies.

## Figures and Tables

**Table 1.** Test results for task1 and task2 using our proposed method

Proposed method	DSC (GTVn)	DSC (GTVp)	Aggregated DSC
	Task1		
SSL-xSLTM-UNet	0.86045	0.7690	**0.8147**
	Task2		
SSL-xSLTM-UNet	0.7731	0.5343	**0.6537**

**Table 2.** Validation results for task1 and task2 using our proposed and state-of-the-art methods

Proposed method	DSC (GTVn)	DSC (GTVp)	Aggregated DSC
	Task1		
3D-ResUNet [15]	0.9345	0.7647	0.8496
xSLTM-UNet	0.9333	0.7752	0.8542
SSL-xSLTM-UNet	0.9194	0.8539	0.8867
	Task2		
3D-ResUNet [15]	0.7589	0.0	0.3794
xSLTM-UNet	0.7684	0.0	0.3842
SSL-xSLTM-UNet	0.8028	0.0	0.4014
